# Intravaginal Progesterone Application as an Efficient and Reproducible Tool for Synchronizing the Mouse Estrous Cycle

**DOI:** 10.1002/rmb2.70027

**Published:** 2026-02-08

**Authors:** Ban Sato, Yutaka Yamashita, Miyuki Shindo, Masahiro Sato, Mitsutoshi Yamada, Kenji Miyado, Natsuko Kawano

**Affiliations:** ^1^ Laboratory of Regulatory Biology, Department of Life Sciences School of Agriculture, Meiji University Kawasaki Kanagawa Japan; ^2^ Division of Laboratory Animal Resources National Research Institute for Child Health and Development Tokyo Japan; ^3^ Department of Genome Medicine National Center for Child Health and Development Tokyo Japan; ^4^ Department of Obstetrics and Gynecology Keio University School of Medicine Tokyo Japan; ^5^ Department of Reproductive Biology National Research Institute for Child Health and Development Tokyo Japan

**Keywords:** animal welfare, intravaginal application, progesterone, pseudopregnant females, synchronization of estrous cycle

## Abstract

**Purpose:**

The synchronization of the estrous cycle is crucial for optimizing reproductive procedures and improving experimental consistency in female mice. Traditional progesterone (P4) administration methods, such as oral or subcutaneous routes, have limitations including invasiveness and variable efficacy. We aimed to develop intravaginal P4 application as a new method for synchronizing the mouse estrous cycle.

**Methods:**

A **c**ustom‐prepared P4 cream was applied to the vagina and washed out the following day. A vaginal smear test was conducted for 4–5 consecutive days. A combination of an anti‐inhibin antibody (AIMA) and P4 was used to improve reproductive performance. The P4‐treated, synchronized female mice were tested to determine whether they could serve as pseudopregnant recipients for embryo transfer (ET).

**Results:**

Intravaginal P4 application effectively synchronized the estrous cycle and did not adversely affect pregnancy rate or litter size. The combined use of AIMA and P4 significantly increased the number of offspring compared to natural mating (*p* = 0.006). Successful offspring production was achieved with improved procedural consistency when this protocol was used to produce pseudopregnant females for ET.

**Conclusions:**

Our method reduces animal discomfort and the need for vasectomized males, offering a practical approach for enhancing reproductive efficiency and animal welfare in research settings.

## 1 Introduction

**FIGURE 1:**
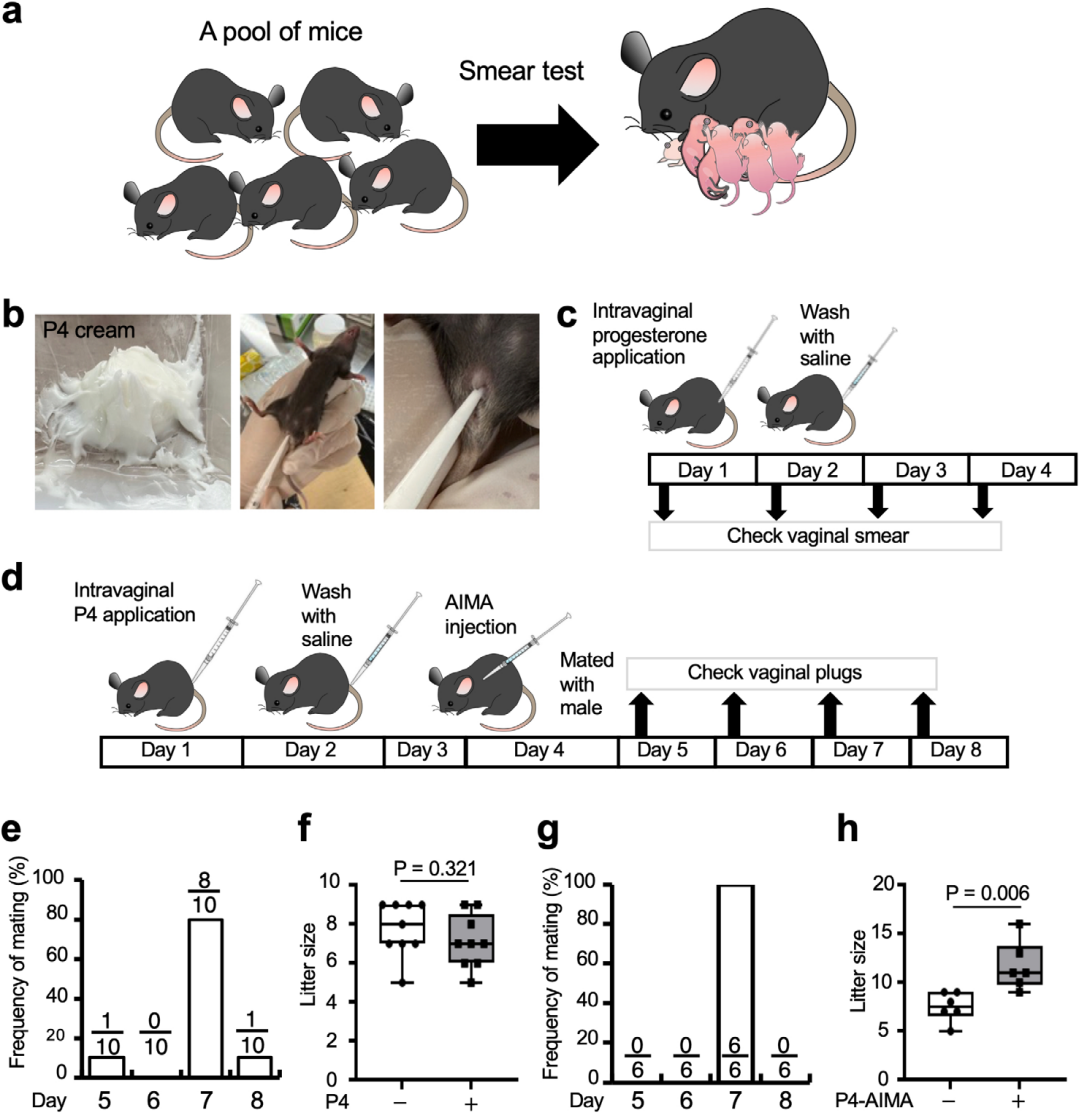
Synchronization of estrous cycles using intravaginal progesterone (P4) application and alteration in the number of offspring following AIMA administration. (a) Schematic illustration of typical method for reproduction. To obtain a single pregnant mouse, a pool of mice and smear tests are necessary. (b) Photographs showing P4 cream preparation and intravaginal P4 application. P4 cream was prepared by mixing powdered P4 with sesame oil (left panel). Tip‐top was first inserted into the vagina and then P4 cream was applied intravaginally (center panel). Enlarged images about tip‐top insertion into vagina (right panel). (c) Schematic representation of the synchronization of estrous cycles. (d) Schematic representation of the mating experiment and subsequent AIMA treatment. (e) Distribution of the day of vaginal plug appearance after mating after intravaginal P4 application. (f) Alterations in the number of offspring using intravaginal P4 application. P4‐; offsprings from natural mating. P4+; offsprings from mating female that synchronized the estrous cycle by P4 application. (g) Distribution of the day of vaginal plug appearance after mating after intravaginal P4 application combined with AIMA treatment. (h) Alteration in the number of offspring with intravaginal P4 application combined with AIMA administration. P4 AIMA‐; offsprings from natural mating. P4‐AIMA +; offsprings from mating female that synchronized the estrous cycle by P4 application and AIMA administration. The data are displayed using box and whisker plots, and statistical analysis was conducted using the Mann–Whitney *U* test. Values within the bars indicate the numbers of females with a vaginal plug of the total number of females tested in each group. AIMA, anti‐inhibin antibody.

Achieving a single pregnancy in mice typically requires four to five mice due to estrous cycle variations; however, this can be minimized using vaginal smear testing and inspection (Figure 1a). Estrous cycle synchronization is a desirable technique in reproductive biology research as it facilitates the alignment of reproductive status among female animals, thereby controlling the timing of ovulation and optimizing the collection of oocytes or embryos for assisted reproductive technologies (ARTs) [1, 2, 3, 4, 5]. By synchronizing cycles, researchers can accurately schedule procedures, such as artificial insemination, embryo transfer (ET), and sample collection, thereby minimizing the variability resulting from natural differences in hormonal and physiological status.

Current methodologies for synchronizing the estrous cycle with progesterone (P4) administration include oral administration, subcutaneous injection, and subcutaneous implantation [6, 7, 8]. Oral administration is generally considered less effective. Although rodents such as mice and rats rarely have a short luteal phase due to an incomplete estrous cycle compared to other mammals that have complete estrous cycles, implant administration methods are not suitable [5]. Recently, several groups have developed a method for synchronizing the estrous cycle by subcutaneous (s.c.) administration of P4 twice [1, 2, 3]. Hasegawa et al. [1, 2, 3] reported that s.c. Administration of P4 synchronized the estrous cycle to an artificial diestrus (luteal phase) by mimicking the presence of a corpus luteum (CL) and applied it to reproductive engineering‐related techniques, such as superovulation, ET, and *improved* genome editing via oviductal nucleic acid delivery (*i*‐GONAD). Although this method has been proven effective for the synchronization of estrous cycles, subcutaneous P4 administration and the use of implants can cause discomfort to animals and require skilled researchers. Therefore, the development of less‐invasive and more accessible methods is desirable.

In rodents such as mice and rats, recipient (foster) females must be in a pseudopregnant state at the time of zygote transfer to ensure the successful implantation and development of embryos [9, 10, 11]. A pseudopregnant mouse for ET is a female mouse produced by mating with a vasectomized (sterile) male mouse or induced by artificial induction via sonic vibration [which is called “easy to get next generation by embryo transfer (EGET)”] [12, 13, 14, 15]. In the process of generating pseudopregnant mice, it is essential to consider that the estrous cycle of female mice recurs every four to 5 days. Consequently, females must be mated multiple times (approximately four times for sufficient stimulation to activate the CL) with vasectomized males because of the unpredictability of the female mouse estrous cycle. To determine the estrous cycle stage (proestrus or estrus), visual inspection of the vaginal condition is routinely conducted, although this procedure requires expertise to accurately interpret subtle visual cues [16, 17]. Therefore, reliable and efficient methods are required to produce pseudopregnant female mice consistently using a predetermined schedule. Several research groups have successfully achieved efficient production of pseudopregnant mice by synchronizing the estrous cycle [3, 18]. However, the success of these methods is contingent on a high level of procedural skill, which highlights the need for a more straightforward approach.

This study aimed to synchronize mouse estrous cycles using intravaginal P4 application (based on a direct application of a **c**ustom‐prepared P4 cream to the vagina) as an easy method to reduce animal discomfort. Using this method, it is possible to confirm mating dates and optimizing ET timing more precisely. By achieving better control over the estrous cycle, researchers can improve the efficiency and success rate of reproductive procedures in mice. Furthermore, successful implementation of this technique could lead to advancements in ARTs and elucidate mammalian reproductive physiology. Intravaginal P4 application could efficiently synchronize the estrous cycle, thereby increasing the number of offspring and facilitating the simple production of pseudopregnant mice. These methodologies are expected to contribute considerably to a reduction in the number of mice used in reproductive engineering‐related experiments.

## 2 Materials and Methods

### 2.1 Animals

C57BL/6N mice (SLC Japan Inc., Shizuoka, Japan), aged 8–12 w, were used for the experiments involving natural mating and superovulation. Female ICR mice (SLC Japan Inc.), aged 9–15 w, were used for embryo collection, natural mating experiments, and as recipients of ET. Male ICR mice (SLC Japan Inc.), aged 10–20 w, were used for natural mating and generation of vasectomized mice. Two‐cell embryos derived from C57BL/6N mice were collected using in vitro fertilization and cultured in potassium simplex optimized medium (KSOM) (Merck Millipore, Burlington, MA, USA) for preparing frozen (vitrified) embryos. Freezing and thawing of 2‐cell embryos were conducted according to the protocol described by Kyudo Co. Ltd. (Saga, Japan).

Mice were housed under specific pathogen‐free conditions. Feed and water were provided *ad libitum*. The animals were maintained in plastic cages in an air‐conditioned (temperature 23°C ± 3°C; humidity 50% ± 10%) and light‐controlled room (illuminated from 05:00 to 19:00). All animal experiments were approved by the Animal Care and Use Committee of the Meiji University (MUIACUC2024‐08). Ethical issues were addressed in accordance with the relevant guidelines and regulations.

### 2.2 Estrous Cycle Monitoring and Synchronization

The estrous cycle of female mice was determined using vaginal smear cytology based on criteria for morphology and cell quantity [16] Figure S1a. The vaginal smears were taken every morning between 10:00 and 11:00 by pipetting with 5 μL of physiological saline. The collected cells were stained using Giemsa stain Merck Millipore.

An amount of 20 μL of custom‐prepared cream of P4 (hereinafter called “P4 cream”) (Merck Millipore; 1 g dissolved in 1 mL sesame oil; Figure 1b and S1b) was applied via intravaginal route to 10 female mice for each estrous stage at noon (11:00–13:00) using a 200‐μL tip. The P4 cream was prepared in small batches and stored in 1 mL syringes (TERUMO, Tokyo, Japan) at room temperature. Each batch was used within 4 weeks of preparation, and no decline in synchronization efficiency was observed during this period. After coating, the vagina was washed out with ~800 μL of physiological saline the following afternoon (16:00–18:00) using a 1000‐μL tip. The treated mice were examined to determine the stage of the estrous cycle on days 2–4 after the intravaginal P4 application (Figure 1b).

### 2.3 Mating Experiments

**FIGURE 2:**
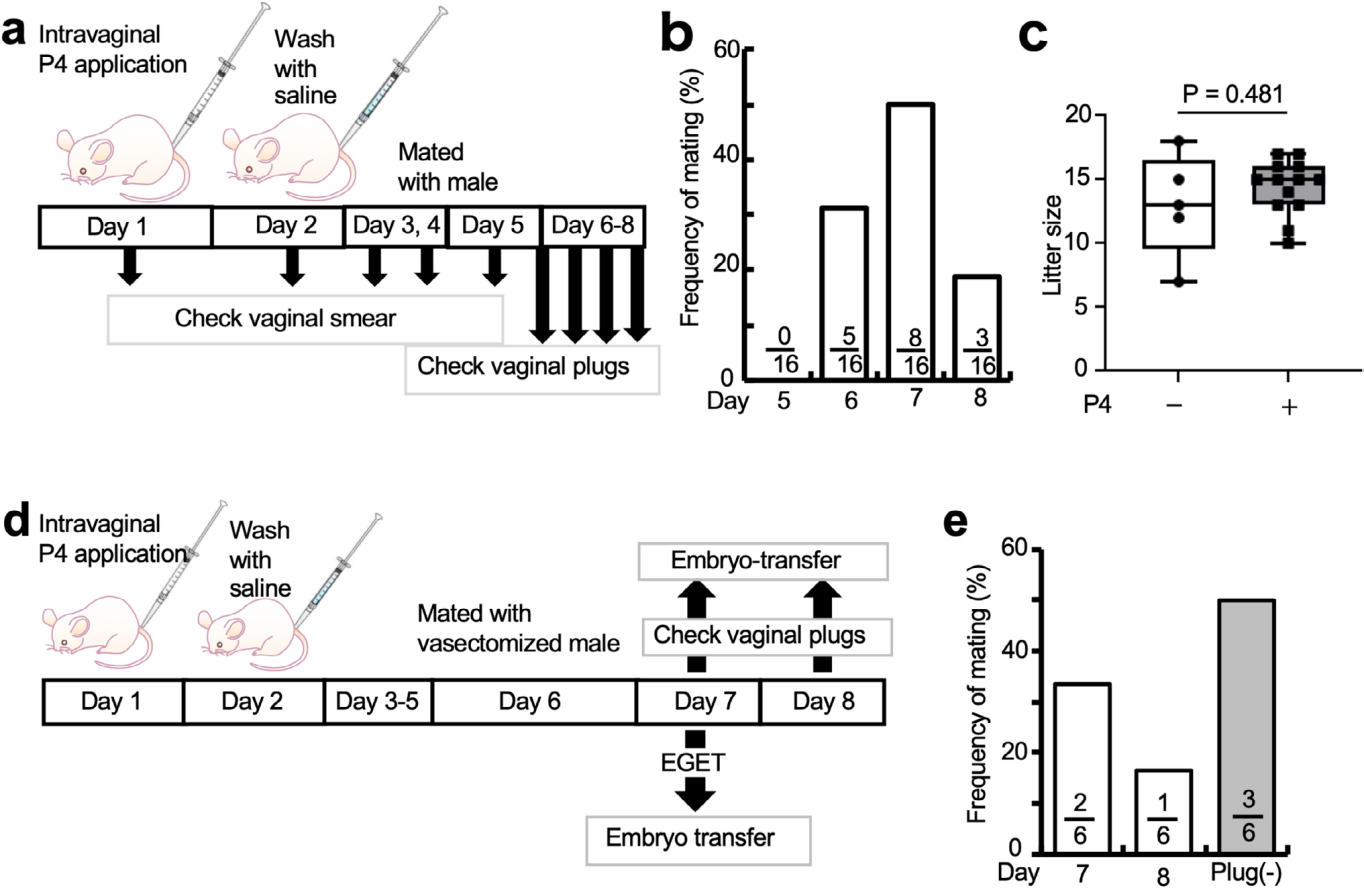
Production of pseudopregnant mice using intravaginal progesterone (P4) application. (a) Schematic representation of the synchronization of estrous cycles. (b) Distribution of the day of vaginal plug appearance after mating after intravaginal P4 application. (c) Alteration in the number of offspring from females with intravaginal P4 application. P4‐; offsprings from natural mating. P4 +; offsprings from mating female that synchronized of the estrous cycle by P4 application. (d) Schematic representation of the production of pseudopregnant mice using intravaginal P4 application. (e) Distribution of the day of vaginal plug appearance after mating with vasectomized mice after intravaginal P4 application. Plug (−) refers to the group in which mating was conducted for two consecutive days, but no plug was observed. The data are displayed using box and whisker plots. Statistical analysis was conducted using the Mann–Whitney *U* test. Values within the bars indicate the numbers of females with a vaginal plug of the total number of females in each group.

Anti‐inhibin monoclonal antibody (5 mg/mL, BioGate Co. Ltd., Gifu, Japan) was used as a superovulation reagent for an increase in offspring [2, 19]. In the mating experiments, AIMA administration (through intraperitoneal (i.p.) injection of 100 μL per mouse) and no administration groups (Figures 1d and 2a) of mice were used with intravaginal P4 cream application to synchronize the estrous cycle.

To determine the appropriate day for mating, a vaginal plug check was conducted over four consecutive mornings until the success of mating (recognized by the presence of vaginal plugs) was confirmed. As a control for the non‐treatment groups, female mice with a swollen, pink, moist, and widely‐open vagina were generally considered to be in the proestrous stage. Selected female mice were placed with males (1:1) overnight to facilitate timed mating. The following morning, the females were checked for the presence of vaginal plugs. The day on which the plugs were detected was defined as day 0 of pregnancy.

After mating, pregnant mice were housed individually and identified based on weight gain and changes in appearance. At 19.5 d post‐mating, fetal viability was assessed by counting live offspring.

### 2.4 Embryo Collection

Superovulation was induced in ICR females using an i.p. injection of 10 IU/body pregnant mare serum gonadotropin (PMSG; ASKA Animal Health Co., Tokyo, Japan), followed by an i.p. injection of 10 IU/body human chorionic gonadotropin (hCG; ASKA Animal Health Co.) 48 h later. The females were mated with males housed in the same cage, and the presence of vaginal plugs was confirmed the following morning. Two‐cell stage embryos were collected by flushing the oviducts with KSOM on day 1.5 d of pregnancy.

### 2.5 Production of Pseudopregnant Mouse by EGET and ET

The P4 cream was applied to the vaginal surface to synchronize the estrous cycle of the mice. Subsequently, pseudopregnant ICR female mice were prepared mating with vasectomized male mice or using EGET (#KN‐595; Natume Seisakusho Co. Ltd., Tokyo, Japan) according to a previously described method [13]. Briefly, the probe was inserted into the vagina of 9–15‐w‐old females in the proestrous stage and stimulation was provided seven times for 30 s, with an interval of more than 30 s between each stimulation at 9:00–10:00 on the day of ET. The female mice in the proestrous phase were selected based on the smear analysis of females that had undergone intravaginal P4 application. Frozen two‐cell embryos derived from C57BL/6N mice were transferred into an oviduct of pseudopregnant mice which mated with a vasectomized male at 14:00–16:00 on the day of ET. Fresh two‐cell embryos derived from ICR mice were transferred into an oviduct of the EGET‐stimulated pseudopregnant mice at 14:00–16:00 on the day of ET. On day 19.5 of pregnancy after ET, the number of viable offspring was determined. In the EGET‐based model, pseudopregnancy was defined as a sustained diestrus‐like vaginal cytology for at least 8 days after EGET stimulation, combined with the ability to support implantation and development of transferred 2‐cell embryos.

### 2.6 Statistical Analysis

Statistical analysis was conducted using GraphPad Prism9 software (GraphPad Software Inc., San Diego, CA, USA) to compare the control and treatment groups. The mating experiments were analyzed using the Mann–Whitney *U* test, following the verification of normal distribution and homogeneity of variance.

## 3 Results

### 3.1 Estrous Cycle Synchronization by Intravaginal P4 Application

To investigate whether the intravaginal application of P4 to 8–12‐w‐old C57BL/6N mice would synchronize their estrous cycles, vaginal smear cytology was first conducted on ten mice at each estrous stage for four consecutive days (Figure S1). On day 1, the P4 cream was administered intravaginally, and on day 2, it was removed using physiological saline (Figure 1b,c). Despite using females at any estrous phase on day 1, 95% of mice tested were synchronized to the diestrous stage when inspection was performed on day 4 (Table 1). This means that our method can successfully induce diestrus in mice by the fourth day of application, regardless of the initial stage of their estrous cycle. Thus, the present intravaginal P4 application offers more expedited and precise initiation of the estrous cycle than conventional techniques.

**TABLE 1:** The transition of estrous stages after intravaginal P4 application in C57BL/6 mice.

Estrous stage^a^	No. (%) of females			
	Day 1	Day 2	Day 3	Day 4
Proestrus	10 (25)	6 (15)	1 (2)	0 (0)
Estrus	10 (25)	10 (25)	4 (10)	0 (0)
Metestrus	10 (25)	14 (35)	15 (38)	2 (5)
Diestrus	10 (25)	10 (25)	20 (50)	38 (95)

^a^ The estrous cycle of female mice was determined using vaginal smear cytology based on criteria for morphology and cell quantity, according to Byers et al. [16].

### 3.2 Intravaginal P4 Application Combined With AIMA Treatment Is an Effective Method for Planned Reproduction

To investigate whether intravaginal P4 application affected pregnancy rates, mating experiments were conducted (Figure 1d). Four days after intravaginal P4 application, the animals were housed with males for 4 d (days 5–8), and the vagina of each female was checked every morning for the presence of a vaginal plug (Figure 1e). There was almost a lack of mating during the periods of 2 d after the start of pairing (days 5–6 after P4 application). By the third day after mating began (day 7), eight of the ten females had mated, representing the highest number observed. Finally, the remaining mice mated on the fourth day (day 8) after P4 application, indicating that 100% of the mice had mated within 4 days of P4 application. All females became pregnant. When the number of offspring was compared between mice that conceived through natural mating and those that received intravaginal P4 treatment, the number of offspring produced by natural mating was comparable to that of the P4 treatment group (Figure 1f). This means that intravaginal P4 treatment does not significantly affect the resulting litter size.

AIMA treatment combined with s.c. P4 administration has been reported to effectively increase the number of offspring using superovulation [2]. Notably, in this study administering AIMA during the diestrous phase was found to be more effective. Based on this finding, we determined to administer AIMA to female mice that had been synchronized to diestrus on day 4 and subsequently the females were mated to males. In the group of P4‐AIMA treatment, 6/6 (100%) females exhibited vaginal plugs on day 7, and 6/6 (100%) became pregnant (Figure 1g). There was no variation in mating dates compared to the control group that received only P4. The number of offspring was 1.6 times higher than that typically produced through natural mating (Figure 1h). An increase in litter size can be expected with AIMA treatment, even in proestrus mice induced using intravaginal P4 application. Thus, the P4 application facilitates the specification of the mating date, thereby enabling control of the delivery date.

### 3.3 Production of Pseudopregnant Mouse Using Intravaginal P4 Application for ET

Since C57BL/6N female mice with synchronized estrous cycles after the treatment with intravaginal P4 application were able to maintain normal pregnancy and litter size, we hypothesized that intravaginal P4 application is also effective for preparation of pseudopregnant ICR females which have been frequently used as foster mothers for generating genetically modified animals. To test this hypothesis, ICR females were subjected to cytological analysis of vaginal smears and mating experiments (Figure 2a). Cytological examination of vaginal smears indicated that when intravaginal P4 application was done on day 1, 18/20 (90%) females exhibited transition to diestrus on day 3, and 14/20 (70%) females reached the proestrous stage on day 5 (Table 2). These results indicate that P4‐mediated synchronization occurred earlier in ICR mice than in C57BL/6N mice. Notably, unlike C57BL/6N mice, ICR females exhibited variation in the mating day. For example, 8/16 females (50%) exhibited successful mating on day 7, while 5/16 females (31%) on day 6 (Figure 2b). All the mice used in the mating experiments became pregnant. This result concurred with that obtained from the cytological analysis of the vaginal smears. The number of offspring obtained from the P4‐treated females was comparable to that obtained from the non‐treated females (Figure 2c). These findings suggest that the intravaginal P4 application protocol is also effective for estrous cycle synchronization in ICR mice. The variability in mating days observed in ICR mice may reflect strain‐specific reproductive characteristics.

**TABLE 2:** The transition of estrous stages after intravaginal P4 application in ICR mice.

Estrous stage^a^	No. (%) of females				
	Day 1	Day 2	Day 3	Day 4	Day 5
Proestrus	6 (30)	2 (10)	0 (0)	2 (10)	14 (70)
Estrus	8 (40)	2 (10)	0 (0)	0 (0)	3 (15)
Metestrus	3 (15)	6 (30)	2 (10)	0 (0)	0 (0)
Diestrus	3 (15)	10 (50)	18 (90)	18 (90)	3 (15)

^a^ The estrous cycle of female mice was determined using vaginal smear cytology based on criteria for morphology and cell quantity, according to Byers et al. [16].

Next, we tested whether ICR females can be used as foster mothers when they are mated to vasectomized males after intravaginal P4 application (Figure 2d). Three of the six (50%) mice exhibited successful mating (as evaluated by the presence of copulation plugs) when they were subjected to mating on days 7–8 (Figure 2e). When ET of cryopreserved 2‐cell embryos was performed towards the P4‐treated pseudopregnant ICR mice on days 7–8, all subjects produced their offspring (31% (15/49)) (Table 3). Thus, the P4‐mediated synchronization technique can be used as a reliable tool for generating foster mothers that are suitable for ET experiments.

**TABLE 3:** Development of 2‐cell embryos transferred to pseudopregnant females after synchronization of estrous stage by P4 application.

Method for induction of pseudopregnancy	Type of embryos	No. of females	No. of pregnant females after ET	No. of embryos transferred	No. (% ± SD) of offspring [litter size]
Mating with vasectomized males	C57BL/6N frozen–thawed	6	3	49	15 (30.8 ± 13.1) [3, 4, 5, 6, 7]
Using EGET	ICR fresh	7	5	77	50 (65.8 ± 19.2) [7, 8, 9, 10, 11, 12, 13]

Abbreviations: EGET, easy to get next generation by embryo transfer; ET, embryo transfer; SD, standard deviation.

Notably, it was reported that pseudopregnancy can be induced by artificial stimulation using sonic vibration instead of mating with vasectomized males, which is called “Easy‐ET method [13].” This vaginal cervical stimulation mimicked a natural mating stimulus and induced pseudopregnancy without the need for vasectomized males. When ET was performed immediately after artificial stimulation, successful implantation and development of offspring were observed, although implantation and birth rates varied depending on the timing of stimulation and embryo stage [13, 20]. Based on these backgrounds, we examined whether the P4‐treated ICR females can be used as foster mothers when they are subjected to EGET‐based artificial stimulation to induce pseudopregnancy. First, we examined whether the pseudopregnant state can be induced in the P4‐treated ICR females after EGET. In the present study, pseudopregnancy in the EGET group was essentially defined in an outcome‐based manner: EGET‐treated females were regarded as functionally pseudopregnant when they later became pregnant and delivered offspring after ET. The smear test indicated that no return to the estrous cycle was observed for at least 8 d after EGET, suggesting successful induction of pseudopregnancy (Figure S2). ET of intact 2‐cell embryos resulted in 5 of 7 recipient females becoming pregnant and generation of viable fetuses with an efficiency of 66% (50/77) (Table 3). These findings are in agreement with the previous notion that EGET‐based induction of pseudopregnancy is superior to the traditional methods based on mating with vasectomized mice, in view of enhanced consistency and reduced variability [20, 21].

## 4 Discussion

### 4.1 The Intravaginal P4 Application as a Newly Developed Method for Synchronizing the Estrous Cycle Offers a Wide Range of Approaches for Developing Reproductive Engineering Technologies in Mice

**FIGURE 3:**
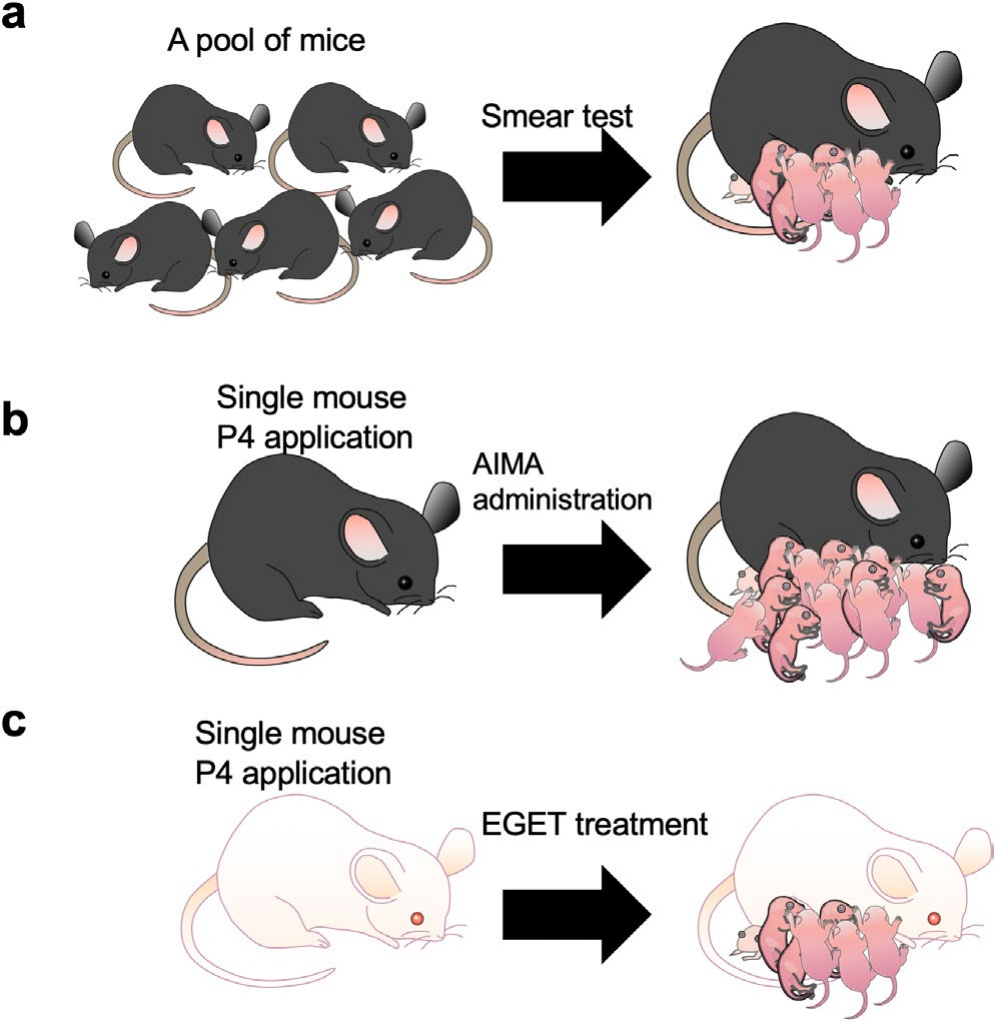
Schematic illustration of intravaginal progesterone (P4) application (a) Traditional method. It always requires smear test to obtain appropriate females that are suitable for mating with males, which are laborious and time‐consuming. (b) Combination of P4 application with AIMA treatment. This treatment allows researchers to proceed their experiments along with precisely scheduled plan to obtain normal number of pups. (c) Combination of P4 application with EGET treatment. This treatment is beneficial for production of pseudopregnant mice along with precisely scheduled plan and does not require vasectomized males, which will contribute to reduce the number of mice used. AIMA, anti‐inhibin antibody; EGET, “easy to get next generation by embryo transfer”.

The intravaginal P4 application method developed in this study offers several advantages over the existing estrous synchronization techniques. Compared to s.c. administration or implants of P4, the present approach is less invasive and causes minimal discomfort to the animals. It also facilitates precise control over the timing of progesterone exposure and removal, enabling more accurate synchronization of estrous cycles in mice (Table 1). The combination of intravaginal P4 application and AIMA treatment has been shown to be particularly effective in increasing litter size (Figure 1h). This approach could be valuable for researchers who aim to maximize offspring production in breeding experiments or have to work with a limited number of animals (Figure 3b). Furthermore, the successful use of this synchronization method was found beneficial for producing pseudopregnant females suitable for ET (Table 3; Figure 3c). By eliminating the need for vasectomized males and facilitating planned production of pseudopregnant recipients, this technique can streamline ET protocols and reduce animal use. Thus, intravaginal P4 application will be one of the less‐invasive, precise, and broadly‐applicable approaches for synchronizing estrous cycles in mice.

### 4.2 Synchronization Efficiency and Mating Outcomes

Our newly developed method for synchronizing the estrous cycle demonstrated a high synchronization rate. Hasegawa et al. achieved successful mating in over 50% of cases using two steps of s.c. injections of P4 [2]. In contrast, in our synchronization method, all the mice (100%) tested were successfully mated, although there were some variations in the exact day of mating Figure 1e. Notably, AIMA administration improved the above‐mentioned variations. As a result, all the mice (100%) tested were mated on the same day, 7 days after P4 application Figure 1g. One possible reason for this high mating rate could be attributed to the ability to wash off progesterone after P4 application. In mice, P4 levels reach their peak shortly after estrous and then gradually decrease [22]. On the second day, the use of a saline solution for washing may have effectively simulated this phase of decline. The present results demonstrate that this method is effective across different mouse strains, including C57BL/6N and ICR mice, suggesting its broad applicability Figures 1e and 2b. The observed differences in synchronization between these two strains will highlight the importance of considering strain‐specific reproductive characteristics when implementing this protocol.

### 4.3 Synchronization of Estrous Cycle for Preparation of Pseudopregnant Females

To create pseudopregnant mice using P4‐based synchronization of the estrous cycle, Hasegawa et al. [3] used vasectomized mice to induce pseudopregnancy. A high rate of plugs (63%) was obtained when the P4‐treated females were subjected to mating to intact males. A similar rate (58%) was also obtained when those females were mated to vasectomized males [3]. In agreement with the findings of Hasegawa et al. [3], we found that 50% of the P4‐treated ICR females tested were successfully mated to vasectomized males (Figure 1e; Table 3).

When EGET was employed instead of mating with vasectomized males, five of seven mice (71%) were pregnant and a large number of offspring (66%) were successfully obtained from the transferred embryos (Table 3). This EGET‐based technique will not only streamline the ET process, but also reduce the necessity of maintaining and managing vasectomized male colonies, which may be potentially beneficial for cost savings and improving animal welfare in research settings. EGET is also beneficial to specify the timing of ET, with which researchers can proceed with their experiments along with a precisely scheduled plan.

### 4.4 Limitations

We here demonstrated the effectiveness of our newly developed method to achieve successful synchronization and pregnancy outcomes. Increasing the sample size in subsequent studies would enhance the statistical power and reliability of the conclusions drawn from the research. Another limitation of the present study is that pseudopregnancy in the EGET group was essentially defined in an outcome‐based manner; EGET‐treated females were regarded as functionally pseudopregnant when they later became pregnant and delivered offspring after ET. This outcome‐based definition differs from the conventional plug‐based confirmation, in which pseudopregnancy can be confirmed by the presence of copulation plugs in females after mating with vasectomized males, making it difficult to compare between these two approaches. Future studies incorporating prospective, standardized criteria, based on the vaginal cytology and hormonal profiling in the EGET‐treated females, are needed to ensure consistency in research across different approaches. Furthermore, further investigation will be warranted to examine the potential long‐term effects on the reproductive health and development of offspring obtained through our system. Future research should focus on optimizing progesterone dosage and exposure time for different mouse strains and age groups. The examination of molecular mechanisms underlying the observed synchronization effects indeed remains a key challenge, especially about the issue concerning why P4 administered vaginally is more effective than oral or subcutaneous routes for estrous synchronization. It is also interesting to test whether the present method can be applied to other rodent species (i.e., rats) commonly used in research.

## 5 Conclusions

Intravaginal P4 application offers a promising new approach for estrous cycle synchronization in female mice. This approach is simpler and more reproducible than the previous approaches based on P4 administration via oral or subcutaneous route. It enables the induction of timely scheduled ovulation, which is beneficial for obtaining a normal number of pups. Furthermore, intravaginal P4 application is useful for obtaining pseudopregnant females according to the precisely scheduled plan. Along with the advances in ARTs, the present technique will become one of the important tools for accelerating reproduction engineering‐related research.

## Funding

This work was supported by Japan Society for the Promotion of Science, 24K1261 and Meiji University.

## Disclosure

Animal Studies: All institutional and national guidelines for the care and use of laboratory animals were followed.

## Conflicts of Interest

The authors declare no conflicts of interest.

## Supporting information


**Figure S1:** Cytology of vaginal smears and preparation for progesterone (P4) cream. (a) Representative images of vaginal smears for each estrous cycle stage. (b) A Photograph showing that 1 g of P4 powder is being weighed. (c) A Photograph showing making of P4 cream, in which powdered P4 is mixed with sesame oil. (d) A Photograph showing dispensing equipment for intravaginal P4 application. The prepared P4 cream was filled into a 1‐ml syringe. A 200‐μl tip was cut along the yellow dashed line and attached to the tip of the syringe.

**Figure S2:** Cytological confirmation of induction of pseudopregnant state by various treatments. (a) Mice in the proestrus (P) and estrus (E) that received EGET treatment did not exhibit a return to estrus by the eighth day. (b) Mice that received only intravaginal progesterone (P4) application exhibited a return to estrus. (c) Mice subjected to both P4 application and EGET treatment did not return to estrus by the eighth day. EGET, easy to get next generation by embryo transfer; M, metestrus; D, diestrus. Each color is a color code used to identify individual animals, and each line shows the estrous cycle transitions for the same individual.

## Data Availability

The data that support the findings of this study are available from the corresponding author upon reasonable request.
