# Supplementary figures and images for: Intravaginal Progesterone Application as an Efficient and Reproducible Tool for Synchronizing the Mouse Estrous Cycle
Source: Reprod Med Biol. 2026 Feb 8;25(1):e70027. doi: 10.1002/rmb2.70027 (PMC12883685; doi:10.1002/rmb2.70027)

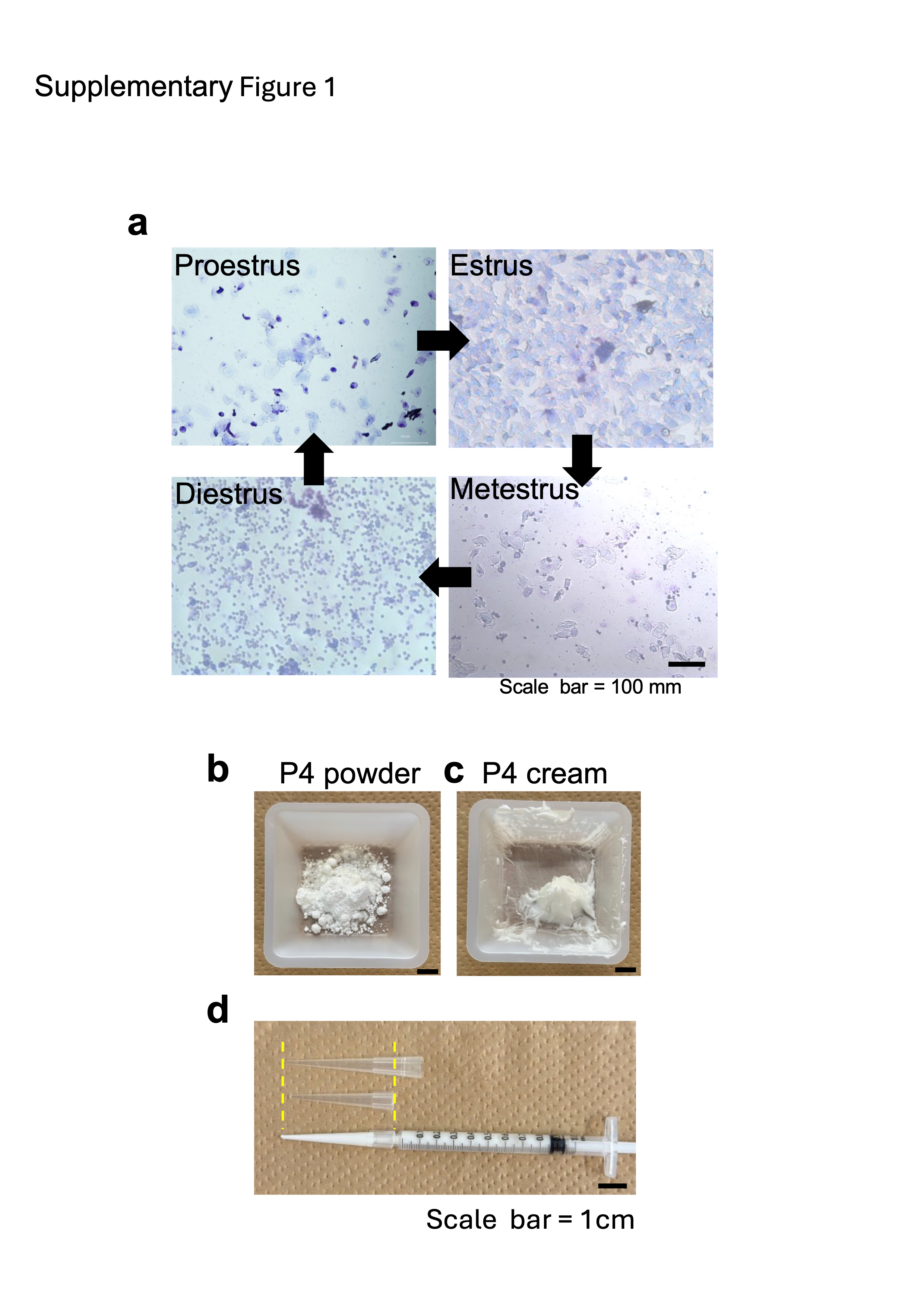

Supplement: Supplementary file 1 — Figure S1: Cytology of vaginal smears and preparation for progesterone (P4) cream. (a) Representative images of vaginal smears for each estrous cycle stage. (b) A Photograph showing that 1 g of P4 powder is being weighed. (c) A Photograph showing making of P4 cream, in which powdered P4 is mixed with sesame oil. (d) A Photograph showing dispensing equipment for intravaginal P4 application. The prepared P4 cream was filled into a 1‐ml syringe. A 200‐μl tip was cut along the yellow dashed line and attached to the tip of the syringe. [file RMB2-25-e70027-s001.jpg]

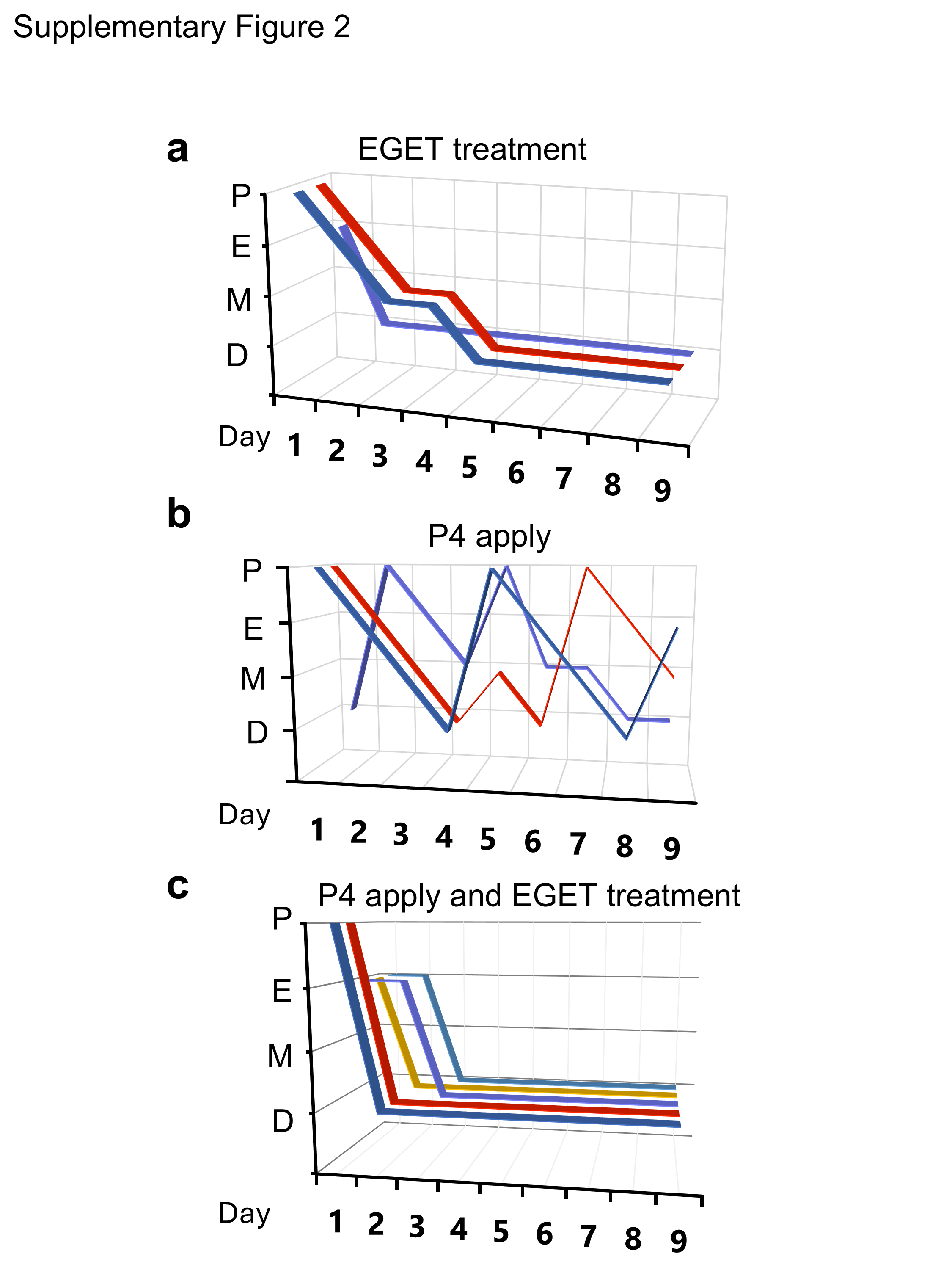

Supplement: Supplementary file 2 — Figure S2: Cytological confirmation of induction of pseudopregnant state by various treatments. (a) Mice in the proestrus (P) and estrus (E) that received EGET treatment did not exhibit a return to estrus by the eighth day. (b) Mice that received only intravaginal progesterone (P4) application exhibited a return to estrus. (c) Mice subjected to both P4 application and EGET treatment did not return to estrus by the eighth day. EGET, easy to get next generation by embryo transfer; M, metestrus; D, diestrus. Each color is a color code used to identify individual animals, and each line shows the estrous cycle transitions for the same individual. [file RMB2-25-e70027-s002.tif]
